# In Vitro and In Vivo Synergetic Radiotherapy with Gold Nanoparticles and Docetaxel for Pancreatic Cancer

**DOI:** 10.3390/pharmaceutics16060713

**Published:** 2024-05-26

**Authors:** Abdulaziz Alhussan, Nolan Jackson, Norman Chow, Ermias Gete, Nicole Wretham, Nancy Dos Santos, Wayne Beckham, Cheryl Duzenli, Devika B. Chithrani

**Affiliations:** 1 Department of Physics and Astronomy, University of Victoria, Victoria, BC V8P 5C2, Canada; 2 Department of Experimental Therapeutics, British Columbia Cancer-Vancouver, Vancouver, BC V5Z IL3, Canada; 3 Radiation Oncology, British Columbia Cancer-Vancouver, Vancouver, BC V5Z 4E6, Canada; 4 Department of Physics and Astronomy, University of British Columbia, Vancouver, BC V6T 1Z1, Canada; 5 Radiation Oncology, British Columbia Cancer-Victoria, Victoria, BC V8R 6V5, Canada; 6 Center for Advanced Materials and Related Technologies, Department of Chemistry, University of Victoria, Victoria, BC V8P 5C2, Canada; 7 Department of Medical Sciences, University of Victoria, Victoria, BC V8P 5C2, Canada; 8 Department of Computer Science, Mathematics, Physics and Statistics, Okanagan Campus, University of British Columbia, Kelowna, BC V1V 1V7, Canada

**Keywords:** gold nanoparticles, docetaxel, radiotherapy, pancreatic cancer, in vivo

## Abstract

This research underscores the potential of combining nanotechnology with conventional therapies in cancer treatment, particularly for challenging cases like pancreatic cancer. We aimed to enhance pancreatic cancer treatment by investigating the synergistic effects of gold nanoparticles (GNPs) and docetaxel (DTX) as potential radiosensitizers in radiotherapy (RT) both in vitro and in vivo, utilizing a MIA PaCa-2 monoculture spheroid model and NRG mice subcutaneously implanted with MIA PaCa-2 cells, respectively. Spheroids were treated with GNPs (7.5 μg/mL), DTX (100 nM), and 2 Gy of RT using a 6 MV linear accelerator. In parallel, mice received treatments of GNPs (2 mg/kg), DTX (6 mg/kg), and 5 Gy of RT (6 MV linear accelerator). In vitro results showed that though RT and DTX reduced spheroid size and increased DNA DSBs, the triple combination of DTX/RT/GNPs led to a significant 48% (*p* = 0.05) decrease in spheroid size and a 45% (*p* = 0.05) increase in DNA DSBs. In vivo results showed a 20% (*p* = 0.05) reduction in tumor growth 20 days post-treatment with (GNPs/RT/DTX) and an increase in mice median survival. The triple combination exhibited a synergistic effect, enhancing anticancer efficacy beyond individual treatments, and thus could be employed to improve radiotherapy and potentially reduce adverse effects.

## 1. Introduction

Pancreatic cancer remains a formidable global health challenge, marked by one of the lowest survival rates among all cancer types, with a five-year survival rate below 10% [1]. The treatment of this cancer depends on its stage and location, generally involving surgery, chemotherapy, and radiotherapy (RT), each presenting specific limitations [2]. For early-stage pancreatic cancer, the optimal treatment often includes surgical resection followed by adjuvant chemotherapy with regimens like FOLFIRINOX or gemcitabine/nab-paclitaxel [3]. However, pancreatic cancer’s aggressive nature and high metastatic potential render many patients ineligible for surgical intervention [4,5]. In chemotherapy, a significant hurdle is achieving effective drug delivery to the tumor. Often, free drugs fail to reach adequate concentrations at the tumor site due to systemic distribution, drug clearance, and the tumor microenvironment (TME) acting as barriers [6]. Radiotherapy is a cornerstone in treating various localized, non-metastatic cancers, including breast, prostate, cervical, head and neck, lung, and brain cancers, with about 50% of cancer patients undergoing RT [7]. Despite this, applying RT to pancreatic cancer is fraught with difficulty due to the organ’s proximity to other vital structures. This proximity limits the radiation dosage for effective local control and raises the risk of damaging nearby healthy tissues, eliciting an urgent need for innovative strategies to overcome these treatment challenges in pancreatic cancer.

Nanotechnology has become a pivotal player in the crusade against the limitations of traditional cancer therapies. This innovative field utilizes nanoparticles (NPs) for targeted drug delivery, capitalizing on both passive and active targeting mechanisms [8]. Passive targeting harnesses tumors’ inherent traits to accumulate NPs, whereas active targeting involves NPs forming specific interactions with molecular targets on tumor cells [9]. Notably, NPs have shown significant promise as radiosensitizers in RT and as vehicles for precise drug delivery in chemotherapy. Gold nanoparticles (GNPs) are a standout in this realm. They can locally enhance the efficacy of RT through their unique photoelectric properties and high X-ray absorption, which leads to increased radiation dose deposition in tumor tissues [10]. Various studies have demonstrated that GNPs can effectively sensitize tumor cells to radiation by generating reactive oxygen species (ROS) and other mechanisms that amplify DNA damage [10,11,12,13,14,15,16,17,18]. Recent advances in nanotechnology have facilitated the development of GNPs that are optimized for better dosing and higher therapeutic effects [13,15,16,18]. Enhancing GNPs as radiosensitizers involves surface modifications with ligands like polyethylene glycol (PEG) for immune evasion and arginyl-glycyl-aspartic acid (RGD) for selective cancer cell targeting [19,20,21]. These features make GNPs efficient in active targeting within the TME. On another front, the chemotherapeutic drug docetaxel (DTX) is an FDA-approved drug for treating cancers like breast, lung, and prostate cancer. DTX can be an effective radiosensitizer in RT [22,23,24]. Despite DTX not being a current treatment for pancreatic cancer, its similarities to nab-paclitaxel, combined with its radiosensitizing properties and compatibility with RT, make it a promising candidate for innovative experiments in this field. The integration of nanotechnology in cancer treatment, particularly through GNPs, opens new avenues for more effective, targeted, and safer cancer therapies.

**Figure 1:**
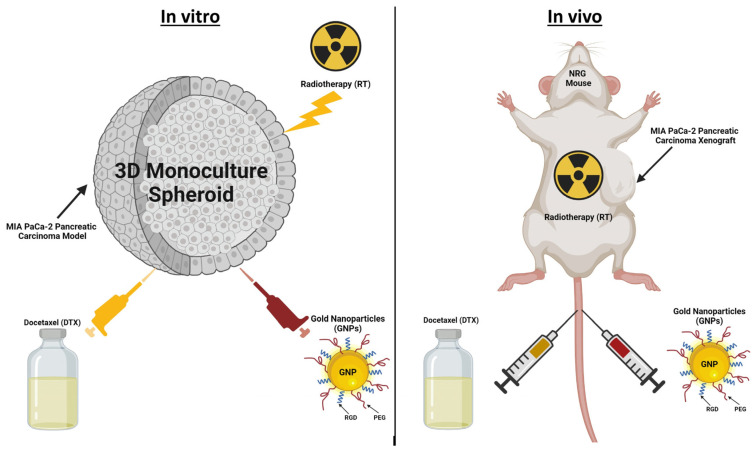
Illustration depicting the integration of nanotechnology, chemotherapy, and radiotherapy (RT) as a combined approach for treating pancreatic cancer, applied both in vitro and in vivo.

In our study, we utilized a 3D in vitro spheroid model and an in vivo model where MIA PaCa-2 pancreatic cancer cells were implanted subcutaneously in NRG mice. These were employed to assess the synergistic effects of a combined treatment regimen consisting of GNPs, RT, and DTX. We anticipate that this tripartite approach—GNPs/RT/DTX—will significantly enhance therapeutic outcomes. This could potentially allow for the reduction of current RT and chemotherapy dosages, thereby sparing normal tissues from excessive exposure. Our investigation focused on evaluating the efficacy of clinically relevant doses of the GNPs/RT/DTX combination (Figure 1). The intent of this study is to unravel the potential benefits of integrating nanotechnology with conventional cancer treatments, aiming to optimize efficacy while minimizing adverse effects on healthy tissues. Using the triple combination of the GNPs/RT/DTX, a 48% and 20% enhancement in tumor control in vitro and in vivo, respectively, was obtained.

## 2. Materials and Methods

### 2.1. Preparation and Analysis of Gold Nanoparticles

Gold nanoparticles (GNPs), approximately 13 nm in size, were prepared using a citrate reduction method [25]. An aqueous solution of chloroauric acid (HAuCl_4_) was prepared, which was then heated to near boiling. Sodium citrate was added to the hot solution, acting as both a reducing agent and a stabilizing agent to prevent nanoparticle agglomeration. This reaction caused the solution to change color from pale yellow to deep red, indicating the formation of colloidal gold nanoparticles. Once the reaction was complete, the solution was cooled, and the gold nanoparticles could be stored, purified, or further processed as required. Visualization of GNPs was achieved through Transmission Electron Microscopy (TEM) using a Ultra-high-resolution Scanning Electron Microscope SU9000 (Hitachi, Pleasanton, CA, USA) as depicted in Figure S1A. These GNPs underwent functionalization with PEG and RGD at specific ratios: one PEG molecule per square nanometer of nanoparticle surface and one RGD molecule for every two PEG molecules. The incorporation of PEG (2000 Da) onto GNPs reduces aggregation and minimizes immune system detection, and RGD (1600 Da) targets the αvβ3 integrin receptor, prevalent in pancreatic cancer cells, enhancing cancer cell specificity [26].

The Perkin Elmer λ 365 UV–Vis spectrophotometer (Waltham, MA, USA) was employed to determine nanoparticle sizes. Figure S1B,C summarize and illustrate the sizes of nanoparticles with PEG and RGD. Furthermore, the stability of these functionalized and non-functionalized nanoparticles was assessed using the LiteSizer 500 particle size analyzer (Anton Paar, Graz, Austria). This analysis determined the ζ potential and particle size distribution, with results summarized in Figure S1B and detailed in Figure S1D,E. These measurements were crucial in demonstrating the nanoparticles’ stability post-functionalization.

### 2.2. Cell Culturing and Spheroid Creation

The MIA PaCa-2 human pancreatic cancer cell line, catalog number CRL-1420™, was procured from the American Type Culture Collection (ATCC). These cells were maintained in high-glucose Dulbecco’s Modified Eagle’s Medium (DMEM; Gibco, ThermoFisher Scientific, Waltham, MA, USA), which was enriched with 10% fetal bovine serum (FBS; Gibco), 1% penicillin/streptomycin (Gibco), and 4 mM GlutaMax (Gibco). For cell detachment, Trypsin-EDTA (Gibco) was used, and paraformaldehyde (PFA; Sigma Aldrich, Oakville, ON, Canada) served for cell fixation. Phosphate-buffered saline (PBS) was utilized for washing the cells. The cells were incubated at a constant temperature of 37 °C in an environment containing 5% CO_2_. For the 3D spheroid cultures, the cells were seeded into ultra-low attachment 96-well microplates (Corning, NY, USA). Each well was populated with 6000 MIA PaCa-2 cells, leading to the formation of spheroids around 400 µm in size. To aid in spheroid formation, the cell medium was supplemented with a 3% Geltrex matrix (Gibco) and kept on ice. These spheroids were then incubated at 37 °C with 5% CO_2_. Experiments with the spheroids began following a 3-day period of incubation, ensuring adequate spheroid formation.

### 2.3. Xenograft Model and Treatments

Female NRG mice were acquired from BC Cancer Research Center Animal Research Center (BCCRC ARC). The MIA PaCa-2 human pancreatic adenocarcinoma cell line was obtained from ATCC (Cat # CRL-1420) in 2021. Cells were started from a frozen vial of lab stock, which was frozen down from the ATCC original vial, tested to be negative for mycoplasma, and kept in lab liquid nitrogen tanks. Cell cultures with passage #3 to #10 and a confluence of 80–90% were harvested for in vivo studies. Cells were grown in DMEM medium supplemented with 2 mM L-glutamine, 10% FBS, and 2.5% Horse Serum at 37 °C in a 5% CO_2_ environment. On study day 0, 5 × 10^6^ tumor cells were implanted subcutaneously into mice in a volume of 100 μL using a 27 G needle. Treatments were administered when the MIA PaCa-2 tumors reached an average of 275–300 mm^3^. Mice were injected with GNPs (2 mg/kg) and free DTX (6 mg/kg) based on their specified treatment using a 28 G needle concurrently and intravenously. The methodology described here has been described, in general, within a service-oriented Animal Care Protocol that has been reviewed and approved by the Institutional Animal Care Committee (IACC) at UBC—protocol #A22-0274. This study protocol will be made available to the IACC on request and under a confidentiality agreement. All data collected and reported on this study protocol will be made available to the IACC on request and under a confidentiality agreement. The care, housing, and use of animals was performed in accordance with the Canadian Council on Animal Care Guidelines.

### 2.4. Gold Nanoparticle Quantification

In the in vitro experiment, spheroids were formed and then concurrently treated with GNPs and DTX, with the GNPs administered at 7.5 μg/mL and DTX at the IC-50 dose of ~100 nM, as determined previously [22]. The treatment lasted for 24 h. Post-treatment, the samples were washed five times with PBS, then incubated in trypsin-EDTA at 37 °C for an hour to disintegrate the spheroids. Cell counts were conducted manually using a hemocytometer. Subsequently, the samples were diluted in 5 mL of Millipore water, treated with 250 μL of aqua regia for every 500 μL of sample, and heated in a mineral oil bath at 90 °C for around 2 h. This was followed by the addition of 100 μL of hydrogen peroxide to each sample, with a further hour in the oil bath. Finally, the samples were diluted with deionized water to achieve a 2.5% *v*/*v* acid concentration.

For the in vivo samples, they were first weighed and blended with 2 mL of TrypLE for breakdown. Then, similar to the in vitro samples, they were diluted in Millipore water, treated with 250 μL aqua regia per 500 μL of sample in a 90 °C mineral oil bath for at least 2 h, and finally diluted to a 2.5% concentration in deionized water. The samples were then filtered through a 0.2-micron filter (Fisher) before the gold content was measured using inductively coupled plasma–mass spectrometry (ICP-MS; Agilent 8800 Triple Quadrupole, Agilent Technologies, Santa Clara, CA, USA), as previously described in our work [24].

### 2.5. Darkfield and Confocal Imaging of Gold Nanoparticles

The 3D spheroids, once formed, underwent treatment with GNPs and DTX as outlined in Section 2.4. These samples were then imaged using a Zeiss LSM 980 confocal microscope with a 20× lens (Carl Zeiss Microscopy GmbH, Jena, Germany). For the imaging process, the spheroids were placed in 35 mm coverslip-bottom dishes (MatTek, Ashland, MA, USA) with minimal media to avoid aspiration and maintain stability. To visualize the gold, the GNPs were tagged with Cy5 fluorescent dye molecules. In the in vivo part of the study, tumor samples fixed in 10% neutral-buffered formalin were processed overnight into paraffin using an automated tissue processor. These were then embedded and sliced into 4 um sections. For darkfield (DF) imaging, the slides were simply covered with a resinous mounting medium. To identify the GNP localization within these in vivo tumor samples, a CytoViva microscope (CytoViva, Auburn, AL, USA) equipped with hyperspectral imaging (HSI) and DF capabilities was employed.

### 2.6. Analysis of Cell Cycle Phases

Post 24 h incubation with GNPs and DTX, the samples were subjected to five PBS washes and then incubated in trypsin-EDTA (Gibco) at 37 °C for an hour to aid in spheroid disintegration. In the in vivo experiment, Collagenase/Dispase (Roche) was used to treat the samples for two hours. Following this, the samples were rinsed with PBS and underwent two rounds of centrifugation at 350× *g* for 5 min each. Subsequently, each sample was washed with 1% PFA and fixed by leaving it at 0 °C for 15 min. After another rinse in PBS and a 5 min centrifugation, the samples were resuspended in 70% ethanol and incubated in the dark at 4 °C for an hour for further fixation. The samples were then filtered using a 100-micron cell strainer and centrifuged at 350× *g* for 10 min at room temperature. This was followed by a rinse in 1 mL of 0.5% bovine serum albumin (BSA) and another centrifugation for 5 min. To degrade RNA, a PBTB (PBS, 0.5% BSA, 0.1% Triton-X 100) solution with RNaseA (100 ug/mL) was used for a 25 min wash at room temperature. Then, propidium iodide (10 μg/mL) was added, and the samples were incubated for another hour before being centrifuged at 350× *g* for 5 min at room temperature. The samples were finally suspended in 1 mL of PBS/BSA and filtered through a 50 μm cell strainer. The last step involved running the samples on a flow cytometer (FACS Calibur, BD Biosciences, Franklin Lakes, NJ, USA) for analysis.

### 2.7. In Vitro and In Vivo Radiation Treatments

One day after administering GNPs to spheroids and before subjecting them to radiation, most of the culture medium was carefully removed, the samples underwent five PBS rinses, and fresh medium was introduced. These samples were then positioned at the center of a Varian TrueBeam linear accelerator, sandwiched between two solid water blocks each measuring 5 cm, located at BC Cancer-Victoria in British Columbia, Canada. They were exposed to 2 Gy of radiation from a 6 MV beam (with a 28 cm × 28 cm field size, 202 monitor units, at a dose rate of 600 monitor units per minute) directed from below. Control samples set to 0 Gy were also brought to the accelerator to ensure the same transport conditions were maintained, though they received no radiation. Additionally, a set of control samples that were not treated with GNPs underwent 2 Gy radiation. The selected thickness of the phantom ensured that each sample received an even dose, along with sufficient material to achieve a complete backscatter dose for precise dose measurement. The 6MV beam was selected for its frequent use in clinical settings. Following the treatments, all samples were transported back to the lab for further analysis through proliferation and immunofluorescence assays to evaluate the treatment outcomes.

For the in vivo treatment, radiation delivery to tumors using linear accelerators was conducted at the BC Cancer Treatment Center in Vancouver, using a single dose of 5 Gy delivered using the high-definition multi-leaf collimator with a 6 MV flattening filter-free photon beam on a Varian TrueBeam^TM^ linear accelerator (at a dose rate of 1400 monitor units per minute). Tumor growth was monitored by measuring tumor dimensions with calipers beginning on the first day of treatment. Tumor length and width measurements were obtained twice a week. Tumor volumes were calculated according to the equation L × W^2^/2, with the length (mm) being the longer axis of the tumor. Tumors were allowed to grow to a maximum size of 800 mm^3^.

### 2.8. Assessment of Cell Proliferation and Spheroid Dimensions

Following the radiation treatment, a cell proliferation assay was carried out on 3D spheroids at 1 and 14 days post-treatment. This was done using CellTiter-Glo 3D (Promega, Madison, WI, USA) and a Biotek Cytation 1 plate reader. To assess the spheroids’ size post-treatment, brightfield images were taken every three days using the 4× objective on the Biotek Cytation 1 plate reader (Agilent Technologies, Santa Clara, CA, USA). These images were used to manually calculate the average diameter of the spheroids with the help of ImageJ software (Java 1.8.0_322 (64-bit)). The area (*A*) of each spheroid was determined by manually tracing its perimeter, and the diameter (*d*) was then calculated using the formula *d* = 2(*A*/*π)*^0.5^. Care was taken to exclude cell debris caused by drug or radiation damage during the outlining process. For each time point, around 8 spheroids were analyzed to establish the average diameter under each condition. To evaluate the potential synergistic effects of GNPs, DTX, and RT, the Bliss independence principle was applied [27]. The model estimates the expected combined effect based on the assumption that each agent targets distinct pathways to exert their anticancer effects, with these pathways being mechanistically independent except for their contribution to the overall response outcome.

The following equation was used:

*E*_combined_ = *E_A_* + *E_B_* + *E_C_* − (*E_A_* × *E_B_* + *E_B_* × *E_C_* + *E_C_* × *E_A_*) +(*E_A_* × *E_B_* × *E_C_*)

where

- *E*_combined_ is the expected combined effect of agents A, B and C;
- *E_A_* is the effect of agent A when used alone;
- *E_B_* is the effect of agent B when used alone;
- *E_C_* is the effect of agent C when used alone.

If the observed effect matches the expected effect calculated using the Bliss model, it suggests that the drugs’ effects are additive. If the observed effect is greater than the expected effect, it suggests that the drugs are synergistic. If the observed effect is less than the expected effect, it suggests antagonism among the drugs.

### 2.9. Immunofluorescence Analysis

Cells were grown on glass coverslips in 6-well dishes. Twenty-four hours post-radiation treatment, the cells underwent a washing process with PBS, followed by a 5 min fixation in 4% paraformaldehyde (PFA). This was succeeded by a PBS rinse and a 20 min incubation in a solution of 2% BSA/0.1% Triton-X. To detect DNA double-strand breaks (DSBs), cells were treated with an optically labeled antibody targeting the repair protein 53BP1. For staining, the primary 53BP1 antibody was diluted at a ratio of 1:200 in a mixture of 0.5% BSA/0.1% Triton-X/PBS. The secondary antibody, in contrast, was diluted at 1:500 in the same solution. The staining procedure began with a one-hour incubation in the primary antibody, followed by a PBS rinse. Next, the cells were washed for 5 min in a solution of 0.5%min BSA/0.175% Tween-20/PBS and then incubated with the secondary antibody for 30 min in the dark. Post-incubation, the cells were washed again with PBS and mounted on glass coverslips using ProLong™ Glass Antifade Mountant and NucBlue™ Fixed Cell ReadyProbes™ Reagent (DAPI) supplied by Invitrogen (Waltham, MA, USA). The assessment of 53BP1 foci, indicative of DSB foci per cell, was performed using confocal microscopy (Zeiss LSM 980) with a 60× oil immersion lens. A minimum of fifty nuclei were examined to measure the number of 53BP1 foci per cell.

## 3. Results and Discussion

### 3.1. Impact of DTX on the Uptake of Gold Nanoparticles In Vitro

In our study, samples were treated with the IC-50 dose of DTX, as established previously [22]. Additionally, the samples received GNPs (approximately 13 nm in diameter) functionalized with PEG and RGD at a low concentration of 7.5 μg/mL to avoid potential toxicity to normal tissues, as noted in references [23,28]. Following a 24 h incubation with GNPs and DTX, the medium was replaced to simulate the depletion of GNPs and drug post a single injection in the body. The uptake of functionalized GNPs by cells occurs through receptor-mediated endocytosis, a mechanism detailed in our earlier work [23]. Subsequently, we processed the samples and quantified the gold content in each cell, as detailed in Section 2.4.

**Figure 2:**
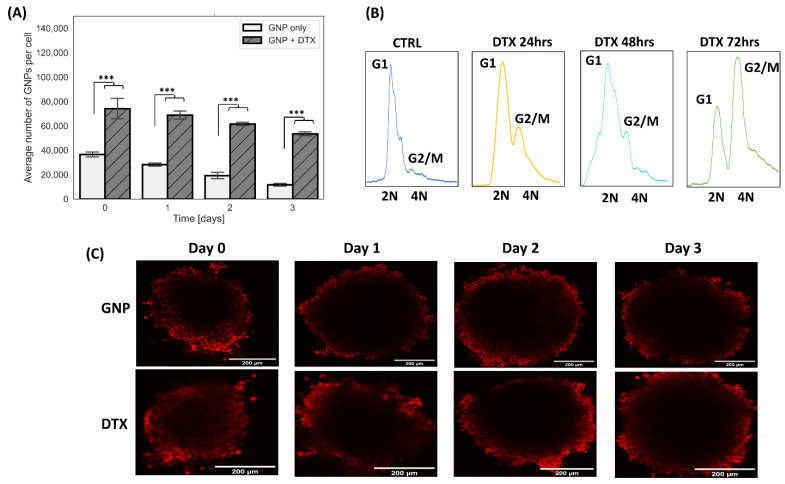
Uptake and retention of gold nanoparticles in pancreatic cancer spheroids. (**A**) The measured uptake of 7.5 µg/mL GNPs in MIA PaCa-2 monoculture spheroids treated with GNPs alone and GNPs combined with DTX as determined by ICP-MS. *** indicates a significance level of *p* < 0.001. (**B**) Cell cycle data for monoculture spheroid cells treated with DTX over a span of 3 days. (**C**) Confocal images showing the uptake and retention of GNPs, depicted in red, over a 3-day period in MIA PaCa-2 monoculture spheroids. These are categorized into three columns: GNPs only (first row) and GNPs plus DTX (second row).

Our results, depicted in Figure 2A, demonstrate increased gold accumulation in cells co-treated with DTX compared to those treated solely with GNPs. Specifically, DTX-treated samples showed a gold increase of 202% for DTX on the first day, retaining 72% of gold, in contrast to 32% in non-DTX-treated samples over 3 days. These findings align with the action mechanism of DTX and other taxane drugs. DTX impedes microtubule function, fostering tubulin polymerization and hindering depolymerization [29,30]. As a result, cell division is disrupted, and cells are arrested in the G2/M phase of the cell cycle, which is notably the most radiosensitive phase. This synchronization in the G2/M phase was observed in cells treated with DTX, with cells displaying a maximum synchronization 72 h post-drug administration (Figure 2B). This inhibition of cell division also promotes GNP accumulation in cells by reducing the GNP distribution to daughter cells, explaining the elevated gold levels in DTX-treated cells (a two-fold increase in amount and retention). In addition, another contributing factor to the increased accumulation and retention can be attributed to the disruption of microtubule dynamics. Microtubules are essential for intracellular transport, facilitating the movement of cellular cargo throughout the cell. DTX’s disruption of microtubule dynamics leads to diminished GNP exocytosis, resulting in increased cellular retention of GNPs. This was also validated by confocal images of spheroids over a 72 h time period (Figure 2C). Interestingly, the images displayed a slight increase in GNP penetration into the spheroid’s core when combined with DTX in comparison to untreated spheroids, suggesting DTX can increase the penetration of GNPs into tumors. This is likely due to the impact of DTX on the extracellular matrix, easing GNPs’ penetration [30,31,32]. The confocal images (Figure 2C) reveal that the uptake and distribution of GNPs within the spheroids are heterogeneous due to differences in spheroid formation. Specifically, cells at the periphery absorb more gold than those at the center, due to the shorter penetration distance required for GNPs to reach peripheral cells [33]. This variability in GNP uptake could influence the radiosensitization effects, as higher concentrations of GNPs within cells are associated with increased radiosensitization [33].

### 3.2. Effects of Gold Nanoparticles, Docetaxel, and Radiotherapy on Spheroid Growth

**Figure 3:**
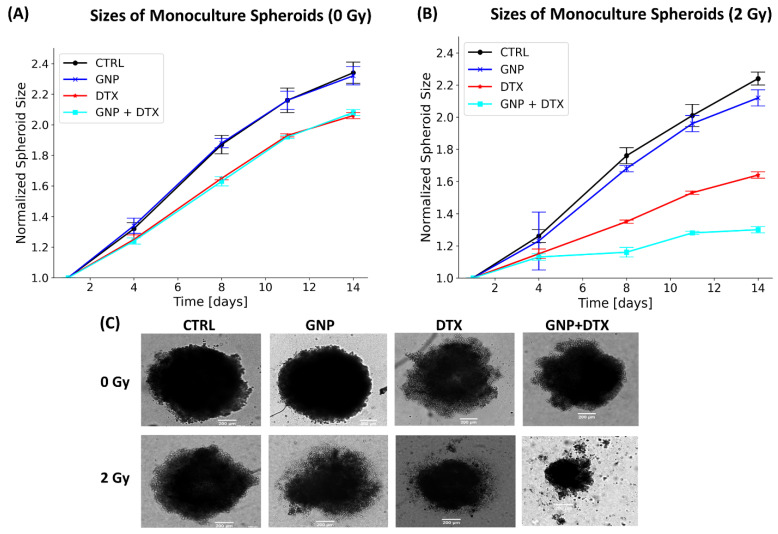
Post-treatment size of monoculture spheroids. (**A**,**B**) Tracking the normalized size of monoculture spheroids across 14 days after treatment, with (**A**) indicating no radiation (0 Gy) and (**B**) showing the effect of 2 Gy radiation. (**C**) Brightfield images of monoculture spheroids captured 14 days following treatment, with a scale bar set to 200 µm.

Following a 24 h incubation with the IC-50 concentration of DTX, the samples were refreshed with new media and then exposed to a single radiation dose of 2 Gy. Figure 3A,B illustrates the spheroids’ diameter changes over a 14-day period post-treatment, both with and without radiation. In the absence of radiation (Figure 3A), spheroid response was as anticipated. For non-irradiated spheroids, GNPs showed no notable impact on size, whereas DTX induced approximately an 11% reduction in spheroid diameter. Conversely, combining GNPs with radiation led to around a 9% reduction in spheroid size. Notably, the combination of GNPs, RT, and DTX resulted in a 41% decrease in size. These outcomes are visually represented in the brightfield images of the spheroids, captured 14 days after treatment, as shown in Figure 3C.

We further validated our results through 3D viability assays, as shown in Figure S2. These assays’ outcomes are overall consistent with the spheroid size measurements presented in Figure 3A–C. They demonstrate the efficacy of DTX, with and without the application of radiation. In the absence of radiation, DTX led to a 45% reduction in cell proliferation. However, when combined with radiation and GNPs, the antiproliferative effect of DTX was enhanced, resulting in a 56% decrease in cell proliferation, as depicted in Figure S2. The proliferation assay was conducted to quantitatively confirm the observations from spheroid size measurements, as relying solely on image-based size estimations might include non-viable cells that contribute to overall size but not to viability. Additionally, due to the non-uniformity of the spheroids, incorporating the proliferation viability assay provides a clearer assessment of the treatment’s effectiveness.

Table 1 illustrates that the triple combination of GNPs/RT/DTX exhibited synergistic effects on reducing spheroid size, whereas the combination of RT/DTX showed additive effects. It is important to note that GNPs alone did not exhibit toxic effects, as evidenced by the unchanged “expected spheroid size” in Table 1 upon the addition of GNPs. Nonetheless, GNPs did manifest synergistic effects in the presence of radiation, although these effects were less pronounced compared to the triple combinations. These observations underscore the effectiveness of the triple combination of GNPs/RT/DTX in reducing cell proliferation and spheroid size.

Due to the most lethal form of DNA damage induced by radiotherapy being DNA double-strand breaks (DSBs), we further investigated the effects of GNPs and DTX by assessing the DNA DSBs exhibited by cells following irradiation by performing an immunofluorescence assay to probe the damage. We measured 53BP1 foci 24 h post-irradiation and visually assessed the average number of DNA DSB foci per cell under various treatment conditions. Confocal images were used to showcase 53BP1 foci in green and nuclei in blue (Figure S4A,B). However, due to the nature of the assay, this was performed using 2D cell cultures rather than 3D tumor spheroids. Upon irradiation, a slight increase in DNA DSBs per cell was noted in cells treated with DTX but without GNPs. However, the combination of GNPs with DTX and radiation led to a marked rise in DNA DSBs (Figure S4). Specifically, GNPs combined with radiation alone resulted in about a 24% increase in DNA DSBs per cell. The increase was approximately 45% when GNPs were used with radiation and DTX, suggesting that the combination of DTX and GNPs as radiosensitizers results in increased DNA damage when combined with radiotherapy. Furthermore, this correlates with the spheroid growth data, in which a significant decrease in spheroid growth was seen in spheroids treated with the triple combination of GNPs/DTX/RT. In contrast, in the absence of radiation, no significant differences in DNA DSBs were observed among the various conditions compared to the control group (Figure S3).

The observed lower experimental spheroid sizes compared to the expected sizes can be largely attributed to the mechanism of action of DTX discussed earlier as well as the radiosensitizing properties of GNPs. When GNPs were combined with DTX, there was an observed increase in GNP accumulation within the spheroids, as detailed in Section 3.1. Additionally, DTX facilitated the synchronization of cells in the G2/M phase, which is known to be the phase of the cell cycle most sensitive to radiation. When GNPs are exposed to ionizing radiation, they engage in the photoelectric effect, interacting with photons to produce secondary electrons [34,35]. These electrons, characterized by their short range and high energy, are potent against cancer cells, inflicting significant damage [36]. They generate additional ionizations and create ROS such as superoxide and hydroxyl radicals. ROS are highly reactive and can cause oxidative stress, damaging vital cellular components like proteins, lipids, and DNA, especially in these radiosensitive cells. This leads to disruption in DNA repair processes and cell cycle progression, culminating in reduced cell viability and enhanced apoptosis [37]. This resulted in the observed synergistic effects.

### 3.3. Impact of DTX on the Uptake of Gold Nanoparticles In Vivo

The in vitro success of the GNP/DTX/RT combination was noteworthy, yet it is well acknowledged that such results do not directly translate to in vivo environments [38]. Transitioning from in vitro to in vivo involves additional considerations [39], and nanomedicine research outcomes in vitro are often more optimistic than what is observed in vivo [40]. Therefore, we meticulously fine-tuned the surface functionality and size of our GNPs using 3D spheroid models prior to in vivo experimentation. For this study, MIA PaCa-2 cells were subcutaneously implanted in NRG mice. We selected GNPs with a 13 nm diameter, functionalized with PEG and RGD peptide, a configuration supported by previous in vitro and in vivo research [20,21,33,41]. Our approach emphasizes several critical factors: intravenous administration at much lower concentrations (mg/kg instead of mg/g), the use of clinically relevant RT energies from a clinical linear accelerator, administering a single injection before RT, and delivering the RT dose one day post NP administration. The chosen dose was 2 mg/kg for GNPs, which is well below the lethal dose (LD_50_) reported for GNPs [42]. The DTX dose of 6 mg/kg approximates a quarter of the weekly human dose of ~75 mg/m^2^ [43,44]. These doses were used to minimize side effects. DTX and GNPs were administered once tumors reached a volume of 275–300 mm^3^.

**Figure 4:**
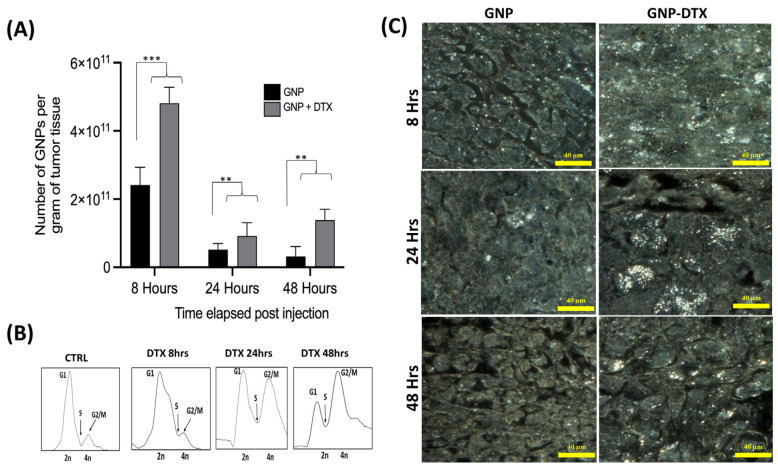
Impact of DTX on in vivo tumor tissues. (**A**) The concentration of GNPs per gram of tumor tissue over time, comparing untreated tissues and those treated with DTX. Statistical significance is denoted as ** for *p* < 0.01, and *** for *p* < 0.001. (**B**) A cell cycle assay showing the changes over time in untreated tumor tissue and tissue treated with DTX. (**C**) Darkfield images of 4 µm sections of tumor tissues. These included untreated tissues and tissues treated with DTX. Scale bar set at 40 µm.

Analysis of GNP uptake in tumor tissues indicated that the tumors in mice treated with DTX had double the GNPs compared to control mice (Figure 4A). Owing to leaky tumor vasculatures and inefficient lymphatic systems, the EPR effect facilitates NP accumulation in tumor cells [6]. However, only 20% of the GNPs remained in the tumor 24 h after treatment. This is likely due to the natural clearance of GNPs from the circulation within this timeframe, reducing the impact of free DTX. Flow cytometry cell cycle data at the 8 h mark showed most tumor cells in the G1 phase (Figure 4B). As exposure to DTX increased, so did GNP accumulation in the tumors, evidenced by a larger fraction of tumor cells synchronized in the G2/M phase at 24–48 h for DTX-treated samples. In contrast, untreated tumors exhibited a further reduction in GNP numbers, attributed to exocytosis and the absence of circulating GNPs after 48 h. These observations are corroborated by darkfield imaging of tumor tissues from untreated and DTX-treated samples (Figure 4C). Visually, there was an apparent increase in GNPs in the tumors treated with DTX compared to untreated ones across different time points, aligning with our quantitative findings.

### 3.4. In Vivo Impact of Gold Nanoparticles, Docetaxel, and Radiotherapy on Tumor Size

Our in vivo findings, depicted in Figure 4, reveal a cell cycle arrest in the G2/M phase concurrent with peak GNP accumulation at 24 h, indicating heightened tumor radiosensitivity at this timepoint. Consequently, a single 5 Gy RT dose was administered at this optimal time using a 6 MV linear accelerator. Tumor progression was monitored until euthanasia at a predetermined endpoint of 800 mm^3^ tumor volume. The treatment groups for this single RT dose included controls (PBS), GNPs, DTX, and GNP/DTX, along with a non-radiated comparison group.

**Figure 5:**
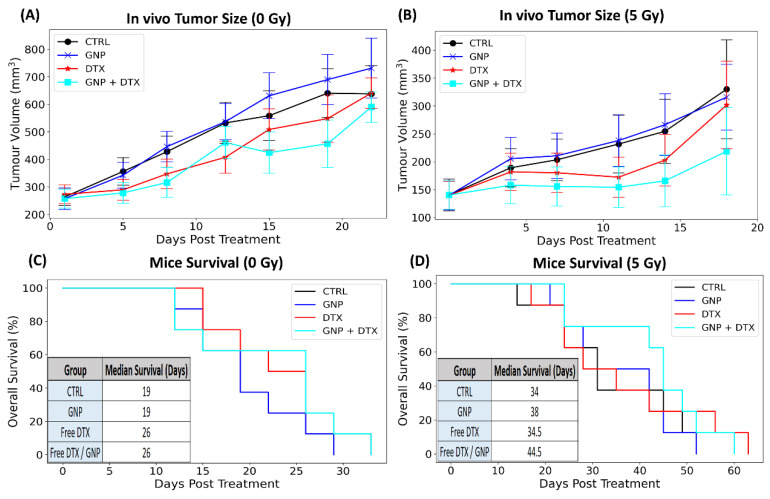
In vivo assessment of GNP/DTX/RT treatment. (**A**,**B**) The normalized tumor volume in mice post-treatment, with (**A**) illustrating results without radiation and (**B**) showing results with radiation. The data, representing an average normalized tumor volume, were obtained from at least five mice and are expressed as mean ± standard deviation, demonstrating the impact of different treatment strategies on tumor growth reduction. (**C**,**D**) The Kaplan–Meier survival curves for female NRG mice inoculated with MiaPaCa2 are shown without radiation (**C**) and with radiation (**D**), comparing the survival rates post-treatment with various strategies. The inset provides the median survival times of the mice in days following tumor incubation.

Figure 5A,B demonstrate tumor volume variations over time post-treatment, with and without radiation. DTX alone did not significantly reduce tumor size, possibly due to the lower dose used, thus limiting its effectiveness despite an observed increase in median mouse survival without radiation (Figure 5C). Additionally, there was no notable difference in tumor volume or mouse survival when comparing groups treated with GNPs to those without, indicating negligible inherent toxicity of GNPs at these low doses without RT. However, the low dose of GNPs meant that the combination of GNPs with RT was not significantly more effective than RT alone. Similarly, the low DTX dose meant that DTX with RT was not significantly more effective than RT alone. It is worth noting that this lack of radiosensitization with GNPs or DTX alone is consistent with our previous findings when using a prostate xenograft model, where the same low concentrations of GNPs and DTX were employed [32], suggesting that an increase in dosage is required to obtain radiosensitization. In the case of GNPs, this is supported by other studies where radiosensitization due to GNPs has been observed at higher doses [45,46]. For instance, early studies by Hainfeld et al. demonstrated a significant increase in long-term mice survival and tumor doubling time due to GNPs when mice were dosed at concentrations a thousand-fold greater in comparison to the mice treated in our experiments [42,47,48]. However, radiosensitizing effects must be achieved at lower concentrations to be clinically viable. A more recent study by Wolfe et al. demonstrated a significant tumor growth delay at a concentration of 10 mg Au/kg when mice were irradiated with a 6 MV photon beam [49]. Yet, this is still five times greater than the concentration in our study, and this could be the reason why GNPs alone did not enhance the radiotherapeutic effects experienced by tumors. Though it is feasible to increase the dosage, doing so could introduce unnecessary toxicity. Rather, combining GNPs with DTX can overcome this. Our data display cooperative benefits in tumor control (*p* = 0.05) of ~ 20% reduction in tumor volume 18 days post-irradiation (Figure 5C) as well as an increase in mice median survival (Figure 5D). Interestingly, this perceived radiosensitization is less than that observed by Wolfe and colleagues. However, it is important to mention a radiation dose of 10 Gy was administered to mice instead of 5 Gy, as used in this study, where this could impact the radiosensitizing effects of GNPs [50]. Nevertheless, our findings suggest that GNP/DTX/RT treatment could be a viable option, demonstrating the potential of nanotechnology to enhance radiotherapy. Incorporating NPs might also allow for lower RT doses, further decreasing toxicity. If translated to clinical settings, the effect of the triple GNP/DTX/RT combination could significantly improve treatment outcomes and potentially increase survival rates, as evidenced by our promising in vivo tumor growth delay and mouse survival data.

### 3.5. Combined GNP, DTX, and RT Treatment Versus Higher Doses of RT Alone In Vitro

**Figure 6:**
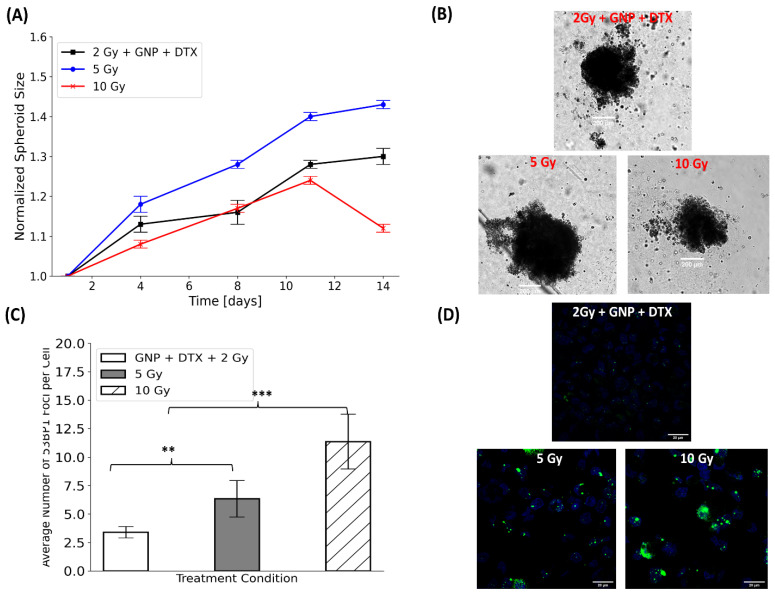
Irradiation effects on monoculture spheroids. (**A**) The size of normalized irradiated monoculture spheroids for 14 days following treatment, providing a longitudinal view of spheroid response to irradiation. (**B**) Brightfield images of these irradiated monoculture spheroids captured 14 days after treatment, with a scale bar of 200 µm. (**C**) Quantifying the average number of 53BP1 foci per cell in irradiated 2D monocultures. Statistical significance is denoted as ** for *p* < 0.01, and *** for *p* < 0.001. (**D**) Confocal microscopy images showcasing the repair protein 53BP1 within the nuclei of MIA PaCa-2 monoculture cells. A scale bar of 20 µm is included for size comparison. In these images, cell nuclei are colored blue, and 53BP1 foci are represented as green dots.

In our study, we evaluated the efficacy of our triple combination therapy (GNPs/2Gy/DTX) against higher doses of RT alone, specifically 5 Gy and 10 Gy. The objective was to determine whether our radiosensitizers (DTX and GNPs) could effectively replace high-dose radiation. Figure 6 presents these findings. Compared to 5 Gy RT, spheroids treated with GNPs/2Gy/DTX were 7% smaller, yet they were 21% larger than those treated with 10 Gy RT (Figure 6A). Brightfield images, taken 14 days post-treatment, qualitatively demonstrate these differences (Figure 6B). An immunofluorescence assay revealed a substantial increase in DNA DSBs for both 5 Gy and 10 Gy treatments compared to GNPs/2Gy/DTX (Figure 6C), with qualitative results depicted in Figure 6D. In terms of cell proliferation (Figure S5), the survival fraction for samples irradiated with 10 Gy was significantly lower than that of the others. However, cell viability in the 5 Gy irradiated samples was comparable to those treated with GNPs/2Gy/DTX. It is crucial to note the role of DTX when combined with RT, as it increases the number of senescent cells [51]. These cells, although heavily damaged, are not entirely non-functional and can still contribute to the metabolic activity of the spheroid, affecting its size and not reflecting the extent of DNA DSB damage. Despite these nuances, our findings are encouraging. They illustrate that the triple combination of GNPs/RT/DTX is comparable to high doses of RT. Given the additional risk of radiation-induced damage to healthy cells with higher radiation doses, our combined treatment modalities using GNPs and DTX with RT could offer a more favorable alternative, achieving better therapeutic outcomes with similar toxicity.

### 3.6. Future Perspective of Gold Nanoparticles in Radiotherapy

RT remains a primary cancer treatment method, used in conjunction with chemotherapy and surgery. However, its application is marred by the toxic effects it can have on healthy tissue, driving the growth of cancer nanomedicine. One strategy to lessen these adverse effects is to integrate radiosensitizers near the tumor to intensify the radiation dose locally. Silver nanoparticles, though also effective in radiosensitization and offering antibacterial properties, may present higher toxicity and stability issues [52]. Hafnium oxide nanoparticles are promising due to their effective dose enhancement and have even reached clinical trials, but they lack the biological functionality and imaging capabilities of GNPs [53]. Gadolinium-based nanoparticles provide dual functionality as MRI contrast agents and radiosensitizers but carry risks of nephrogenic systemic fibrosis, especially in patients with renal issues, and are complex to synthesize [17]. In comparison, GNPs offer superior biocompatibility, functional versatility, and dual functionality, making them one of the most promising options for future cancer therapies. GNPs are notable for their high atomic number, which enhances radiation absorption, leading to increased ROS production, which damages cancer cell DNA. GNPs have shown promise in enhancing radiation effects in lab experiments, especially when combined with other radiosensitizing chemotherapeutic drugs, but they need further development for widespread clinical use. Currently, there are few GNP formulations in clinical trials for cancer, with none being used for radiosensitization [54]. Nonetheless, GNPs have proven effective in increasing radiosensitivity in various cell and animal studies. The challenge lies in the variability of GNPs’ physical properties, which affects their clinical applicability. Combining GNPs with established radiosensitizers could improve the current RT approaches. GNPs have significant potential to act as precise delivery systems for radiosensitizing agents, potentially revolutionizing cancer therapy. Agents such as taxane drugs and other chemotherapeutic drugs are seen as viable candidates for pairing with GNPs and RT to achieve enhanced tumor-targeting effects.

## 4. Conclusions

To assess the efficacy of combining gold nanoparticles (GNPs), radiotherapy (RT), and docetaxel (DTX), we employed an in vivo xenograft model and in vitro spheroid model of pancreatic cancer cells. Our research highlights the significant therapeutic advantages of using GNPs and DTX in tandem with RT, outperforming RT used alone. In this innovative approach, GNPs, functionalized with PEG and RGD, serve as efficient radiosensitizers. They amplify RT’s effectiveness by generating ROS that inflict damage on tumor cells. Simultaneously, DTX plays a role in augmenting the uptake and retention of GNPs in cells and synchronizing them into the most radiosensitive phase of the cell cycle. The combination of GNPs, DTX, and RT produces a comprehensive attack on tumors, causing DNA damage and cell cycle arrest. This synergy results in a marked decrease in cell survival and spheroid size in vitro, pointing to enhanced radiosensitivity. Furthermore, in vivo results displayed a significant decrease in tumor size and increase mice survival for mice treated with GNPs/RT/DTX. Our findings suggest that incorporating radiosensitizers could lower the required RT and chemotherapy dose for effective treatment, thus potentially reducing radiation-induced harm and chemotherapy side effects. This study underscores the potential of a synergistic approach combining nanotechnology and RT in cancer therapy.

## Figures and Tables

**Table 1:** Contrast between experimentally measured spheroid size changes and predicted values based on the Bliss independence model. Signifies statistical significance * at *p* < 0.05 and *** at *p* < 0.001.

	Expected Spheroid Size	Experimental Spheroid Size	Combined Effect
**GNPs/RT/DTX**	45.70% ± 2.57%	39.59% ± 2.08%	Synergistic ***
**RT/DTX**	45.07% ± 2.57%	45.85% ± 2.25%	Additive *
**GNPs/RT**	91.07% ± 5.01%	75.87% ± 3.42%	Synergistic ***

## Data Availability

Datasets generated and/or analyzed during the current study are available from the corresponding author on reasonable request.

## Supplementary Materials

The following supporting information can be downloaded at: https://www.mdpi.com/article/10.3390/pharmaceutics16060713/s1, Figure S1: Gold nanoparticles (GNPs) characterization; Figure S2: Monoculture spheroids proliferation post-treatment; Figure S3: 2D monoculture repair protein mapping; Figure S4: 2D monoculture repair protein mapping; Figure S5: Monoculture spheroids proliferation post-treatment.
