# Supplementary material for: In Vitro and In Vivo Synergetic Radiotherapy with Gold Nanoparticles and Docetaxel for Pancreatic Cancer
Source: Pharmaceutics. 2024 May 26;16(6):713. doi: 10.3390/pharmaceutics16060713 (PMC11206706; doi:10.3390/pharmaceutics16060713)
Supplement: Supplementary file 1 [file pharmaceutics-16-00713-s001.zip › pharmaceutics-2973431-supplementary.pdf]

In Vitro and *In Vivo* Synergetic Radiotherapy with Gold Nanoparticles and Docetaxel for Pancreatic Cancer

Supplementary Section S1: GNPs Characterization

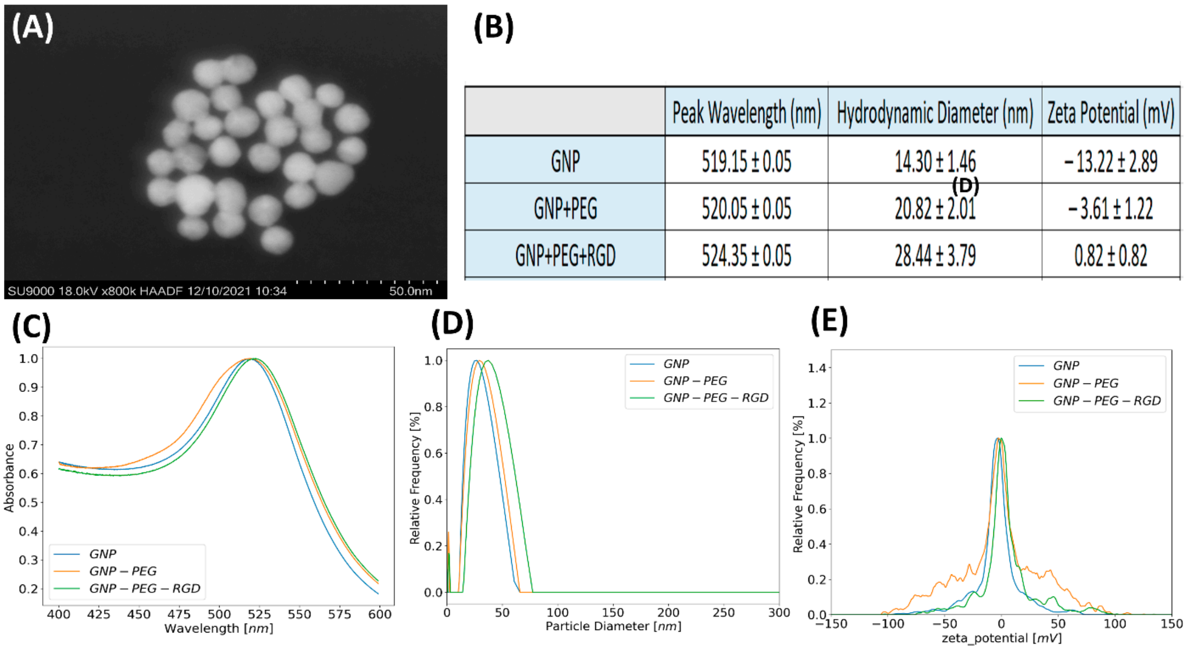

	Peak Wavelength (nm)	Hydrodynamic Diameter (nm)	Zeta Potential (mV)
GNP	519.15 ± 0.05	14.30 ± 1.46	−13.22 ± 2.89
GNP+PEG	520.05 ± 0.05	20.82 ± 2.01	−3.61 ± 1.22
GNP+PEG+RGD	524.35 ± 0.05	28.44 ± 3.79	0.82 ± 0.82

Figure S1. Gold nanoparticles (GNPs) characterization. **(A)** High-Angle Annular Darkfield (HAADF) Scanning Transmission Electron Microscopy (STEM) image of GNPs **(B)** A table outlines the peak absorption wavelength, hydrodynamic diameter, and mean ζ-potential for GNPs, GNPPEG, and GNPPEG-RGD. **(C)** UV–visible absorption spectra of GNPs, GNPPEG, and GNPPEG-RGD. **(D)** The hydrodynamic diameter of GNPs, GNPPEG, and GNPPEG-RGD. **(E)** The ζ-potential of GNPs, GNPPEG, and GNPPEG-RGD.

Supplementary Section S2: Cell Proliferation

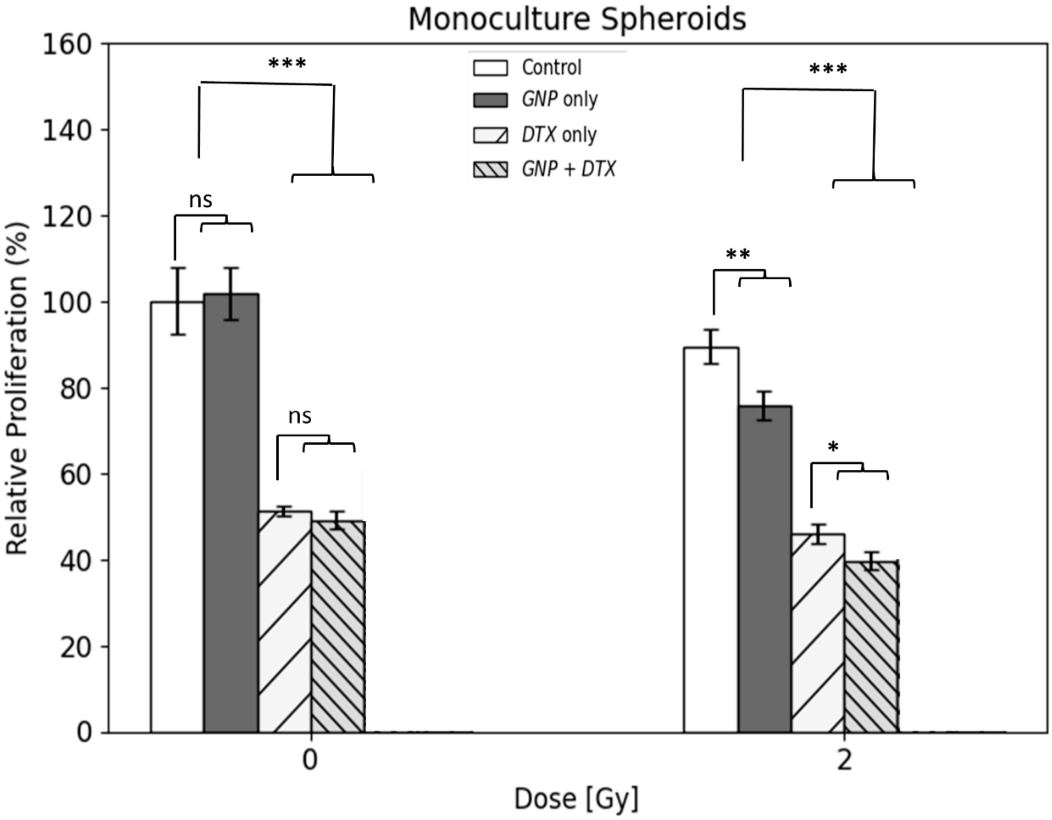

Figure S2. Monoculture spheroids proliferation post-treatment. Monoculture spheroids relative cell proliferation at day 14 post-treatment. ns indicates non-significance, * indicates $p < 0.05$, ** indicates $p < 0.01$, *** indicates $p < 0.001$.

Supplementary Section S3: DNA DSB no Radiation

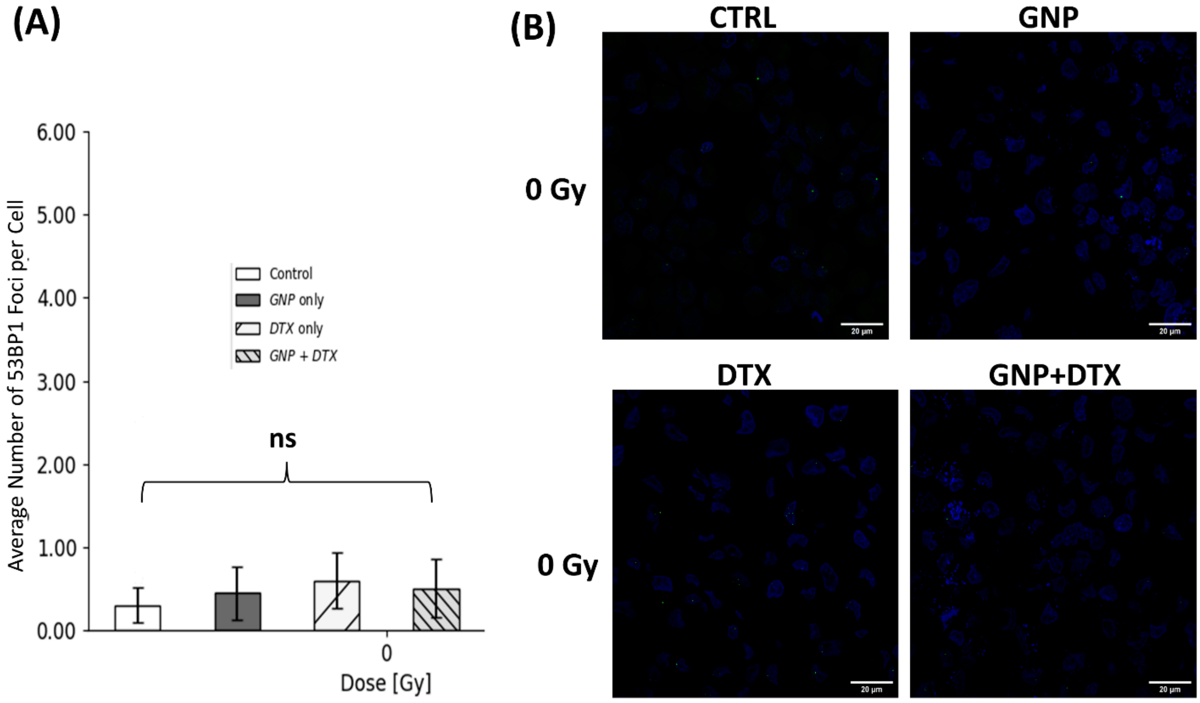

Figure S3. 2D monoculture repair protein mapping. **(A)** The average number of 53BP1 per cell in 2D monoculture without radiation following treatments with different agents**.** ns indicates non-significance, * indicates p < 0.05, ** indicates p < 0.01. **(B)** Confocal microscopy images of repair protein 53BP1 in the nucleus of non-irradiated monocultures of MIA PaCa-2. The cell nuclei are stained blue, while the green dots indicate 53BP1 foci. Scale bar: 20 µm.

Supplementary Section S4: DNA DSB with Radiation

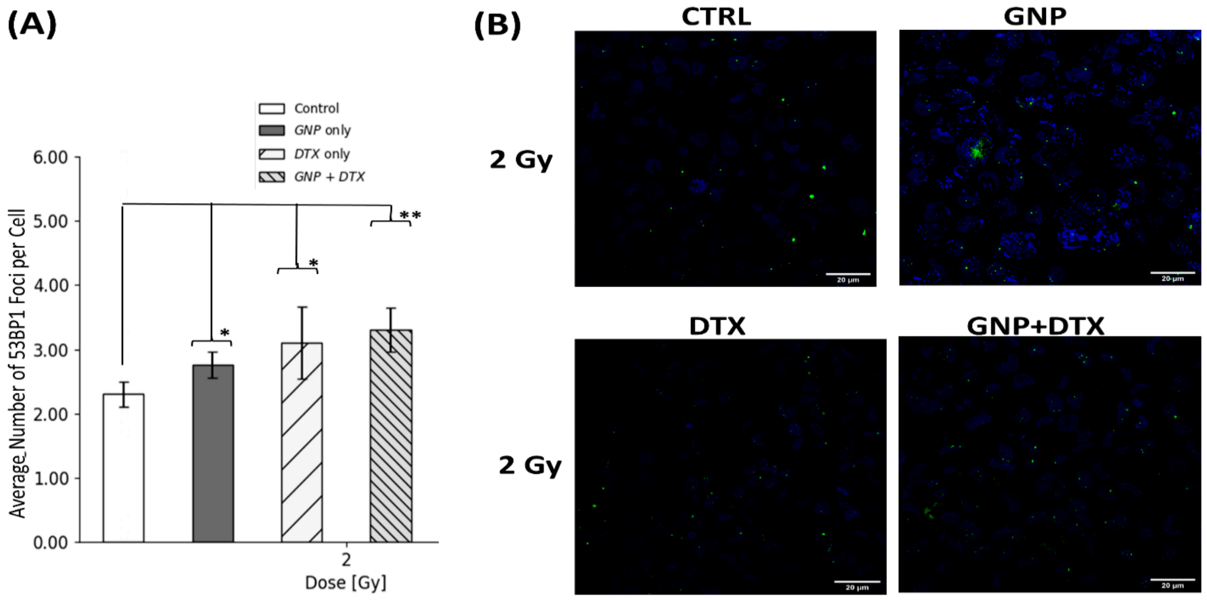

Figure S4. 2D monoculture repair protein mapping. **(A)** The average number of 53BP1 per cell in 2D monoculture 24 hours after irradiation with a dose of 2 Gy following treatments with different agents**.** ns indicates non-significance, * indicates $p < 0.05$, ** indicates $p < 0.01$. **(B)** Confocal microscopy images of repair protein 53BP1 in the nucleus of non-irradiated monocultures of MIA PaCa-2. The cell nuclei are stained blue, while the green dots indicate 53BP1 foci. Scale bar: 20 µm.

Supplementary Section S5: Proliferation GNPs & DTX with RT vs 5/10 Gy

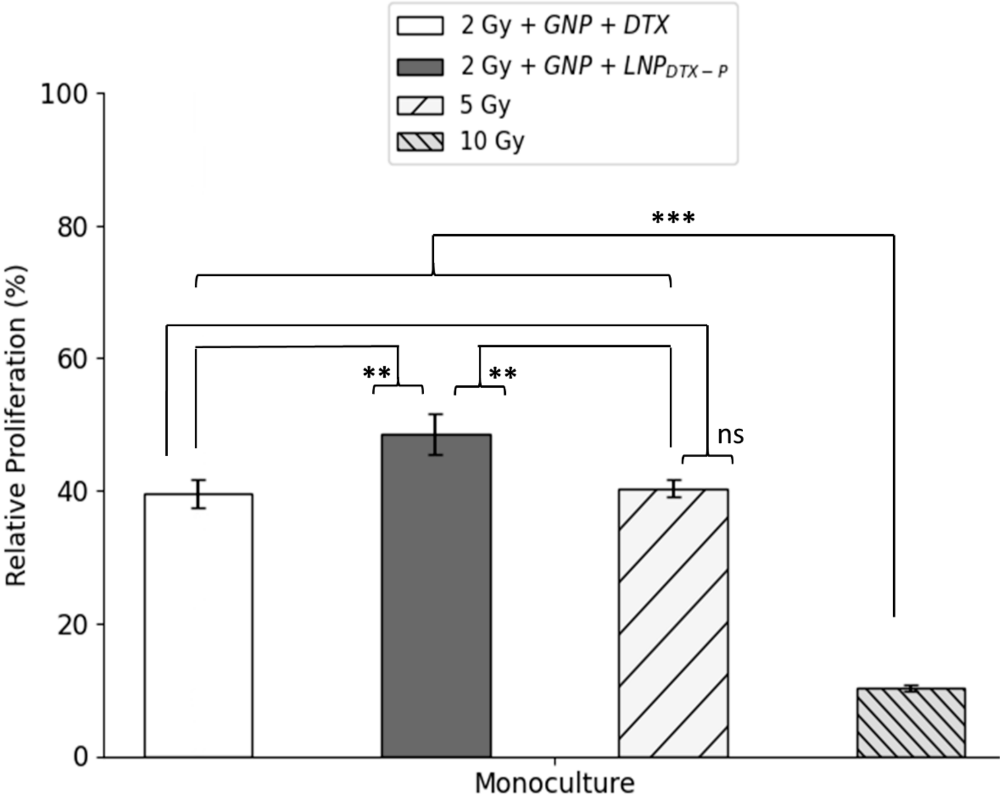

Figure S5. Monoculture spheroids proliferation post-treatment. Monoculture spheroids relative cell proliferation at day 14 post-treatment. ns indicates non-significance, ** indicates $p < 0.01$, *** indicates $p < 0.001$.
